# Möglichkeit von Prüfungsszenarien unter Pandemiebedingungen

**DOI:** 10.1007/s00106-024-01422-1

**Published:** 2024-02-02

**Authors:** T. F. Jakob, P. Maier, A. Knopf, A. K. Rauch, C. Offergeld, T. Hildenbrand

**Affiliations:** 1 Department of Oto-Rhino-Laryngology, Medical Center – University of Freiburg, Faculty of Medicine, University of Freiburg, Killianstr. 5, 79106 Freiburg, Deutschland; 2 Eye Center, Medical Center – University of Freiburg, Faculty of Medicine, University of Freiburg, Killianstr. 5, 79106 Freiburg, Deutschland

**Keywords:** Medizinische Lehre, COVID-19, Pandemien, HNO-Heilkunde, OSCE, Medical education, COVID-19, Pandemics, Otorhinolaryngology, OSCE

## Abstract

**Hintergrund:**

Die COVID-19-Pandemie verursachte weltweit Kontaktbeschränkungen, mit Auswirkungen auch auf das Medizinstudium. Da keine Präsenzveranstaltungen möglich waren, musste innerhalb kürzester Zeit ein digitales Curriculum erstellt werden. Die Rahmenbedingungen für ein Assessment stellten ein noch bedeutenderes Problem dar. Für Prüfungen wie die „objective structured clinical examinations“ (OSCE) mussten Lösungen gefunden werden, da die Durchführung in manchen Bundesländern sogar explizit verboten war. Ziel dieser Arbeit war die Prüfung der Durchführbarkeit einer OSCE unter Pandemiebedingungen.

**Material und Methoden:**

Am Ende des Sommersemesters 2020 absolvierten 170 Studierende eine kombinierte HNO- und augenheilkundliche OSCE. Die Prüfung fand unter strenger Beachtung der Hygieneauflagen über 5 Tage statt. Während das HNO-Konzept virtuell ausgerichtet war, fand die augenheilkundliche Prüfung als Präsenzprüfung statt. Im Anschluss erfolgte die Bewertung der OSCE durch die Studierenden.

**Ergebnisse:**

Zwischen 106 und 118 der Studierenden antworteten auf die jeweiligen Fragen. Im Vergleich der Präsenz- mit der virtuellen OSCE bevorzugten etwa 49 % die Präsenz-OSCE und etwa 17 % die virtuelle OSCE, etwa 34 % fanden beide Varianten gleich gut. Insgesamt wurde die Kombination aus HNO- und augenheilkundlicher OSCE als positiv gewertet.

**Schlussfolgerung:**

Auch unter Pandemiebedingungen ist das Abhalten einer OSCE möglich. Für eine optimale Vorbereitung der Studierenden bedarf es u. a. einer Umstellung der Lehre auf ein digitales Curriculum. Die Kombination aus HNO- und augenheilkundlicher OSCE wurde von den Studierenden positiv bewertet, wobei die Präsenz-OSCE bevorzugt wurde. Bei insgesamt hoher Zufriedenheit auf studentischer Seite zeigt sich die Machbarkeit einer virtuellen Prüfung bei detaillierter und gut geplanter Vorbereitung.

## Abstract (en)

**Background:**

Due to the COVID-19 pandemic, contact restrictions occurred worldwide, which affected medical schools as well. It was not possible to hold classroom lectures. Teaching contents had to be converted to a digital curriculum within a very short time. Conditions for assessments posed an even greater challenge. For example, solutions had to be found for objective structured clinical examinations (OSCE), which were explicitly forbidden in some German states. The aim of this study was to evaluate the feasibility of an OSCE under pandemic conditions.

**Materials and methods:**

At the end of the 2020 summer semester, 170 students completed a combined otolaryngology and ophthalmology OSCE. Examinations were held in small groups over the course of 5 days and complied with strict hygiene regulations. The ophthalmology exam was conducted face to face, and the ENT OSCE virtually. Students were asked to rate the OSCE afterwards.

**Results:**

Between 106 and 118 of the students answered the questions. Comparing the face-to-face OSCE with the virtual OSCE, about 49% preferred the face-to-face OSCE and 17% preferred the virtual OSCE; 34% found both variants equally good. Overall, the combination of an ENT and ophthalmology OSCE was rated as positive.

**Conclusion:**

It is possible to hold an OSCE even under pandemic conditions. For optimal preparation of the students, among other things, it is necessary to transform teaching contents to a digital curriculum. The combination of an ENT and ophthalmology OSCE was positively evaluated by the students, although the face-to-face OSCE was preferred. The overall high satisfaction of the students confirms the feasibility of a virtual examination with detailed and well-planned preparation.

## Prüfung praktischer Fertigkeiten und klinischer Kompetenzen

Eine „objective structured clinical examination“ (OSCE) ist ein Prüfungsformat, welches in der medizinischen Ausbildung zur Anwendung kommt. Diese Form der Prüfung wurde bereits 1975 von Harden [8] und 1979 ausführlicher [7] beschrieben. Das Ziel ist die Prüfung von praktischen Fertigkeiten und klinischen Kompetenzen und nicht nur das Abfragen von Faktenwissen, wie es in mündlichen oder schriftlichen Prüfungen üblich ist [3]. Die Studierenden durchlaufen einen Parcours mit einer bestimmten Anzahl von Prüfungsstationen, die unterschiedliche Aufgaben enthalten [7]. Die Aufgaben können Anamneseerhebung, körperliche Untersuchungen und Arbeiten an Modellen, Computern oder mit Videos beinhalten. Des Weiteren sind Stationen mit standardisierten Patient*innen möglich [3]. In der Studienordnung der Albert-Ludwigs-Universität Freiburg für den Studiengang Humanmedizin vom 22.02.2012 mit der letzten Änderung vom 11.11.2015 sind in den Fächern Augenheilkunde, Chirurgie, Hals-Nasen-Ohren-Heilkunde, Neurologie und Palliativmedizin OSCE-Prüfungen vorgesehen.

Durch die Coronavirus-Pandemie kam es im Zuge der Kontaktbeschränkungen auch zu Auswirkungen auf die Lehre der Medizinstudierenden [30]. Bereits im Sommersemester 2020 gab es positive Beispiele der Universitäts-HNO-Kliniken für die Umwandlung der Präsenzlehre in ein virtuelles Semester [21, 22, 31]. So wurde die Lehre in der Universitäts-HNO-Klinik Freiburg in ein komplett digitales HNO-Curriculum mit Podcasts, Videos, Tutorials, Videokonferenzen, webbasierten Lernprogrammen und internetgestützten Lernplattformen transformiert. Klinische Fertigkeiten, wie z. B. die HNO-Spiegeluntersuchung [12] oder die Koniotomie, wurden virtuell mithilfe der 4‑Schritt-Methode nach Peyton vermittelt [17]. Bei dieser Lehrmethode werden in einem ersten Durchgang die Abfolgen der zu vermittelnden Tätigkeit in Einzelschritte unterteilt und in normaler Geschwindigkeit vom Lehrenden vorgeführt („Demonstration“). Im zweiten Durchgang werden die Schritte entsprechend der vorgegebenen Abfolge langsam wiederholt und kommentiert („Dekonstruktion“). Im dritten Durchgang lässt sich der Lehrende die einzelnen Schritte vom Studierenden erklären und führt sie unter Anleitung des Studierenden aus („Verständnis“). Im vierten Durchgang führen die Studierenden die Schritte komplett eigenständig durch und erklären diese („Durchführung“). Schritt 3 konnte als wichtigster Schritt identifiziert werden [10, 13]. Dies wurde auf die Kombination der bildlichen Vorstellung des Handlungsablaufs, gekoppelt mit verbaler Beschreibung der motorischen Tätigkeit und der Ausführung einer Fertigkeit zurückgeführt. Es konnte gezeigt werden, dass diese Lehrmethode in Bezug auf Professionalität und Arzt-Patienten-Kommunikation im Vergleich zu Standardanweisungen gleichwertig oder sogar überlegen ist und zu einer schnelleren Verinnerlichung führt, wenn die Studierenden die erlernte Fertigkeit zum ersten Mal ausführen [13]. Diverse Modifikationen wurden entwickelt, um die Methode für die virtuelle Lehre, das Blended Learning und für die Lehre in Gruppen zu adaptieren [15, 19, 20, 29].

An der Albert-Ludwigs-Universität Freiburg absolvieren Studierende des 7. und 8. Semesters verschiedene Blockpraktika, u. a. je ein zweiwöchiges Blockpraktikum in der HNO- und in der Augenheilkunde. Am Ende des jeweiligen Blocks fand bislang eine entsprechende OSCE-Prüfung statt. Sowohl im Sommer- als auch Wintersemester wurden Lehreinheiten über 14 Wochen verteilt in 7 Blöcken mit jeweils etwa 25 Studierenden durchgeführt.

Da die Absolvierung einer OSCE in der HNO- und Augenheilkunde in der Prüfungsordnung vorgeschrieben ist, musste in der Pandemie schlagartig ein Prüfungsablauf realisiert werden, welcher mit den Hygieneauflagen zu vereinbaren war.

Ziel dieser Arbeit war die Etablierung einer digitalen OSCE und die Evaluation durch die Studierenden. Durch Integration einer augenheilkundlichen OSCE, welche mit Prüfern vor Ort stattfand, konnten die beiden Prüfungsformate (virtuelle OSCE vs. Präsenz-OSCE) im weiteren Sinne direkt verglichen werden.

## Material und Methoden

### Vorbereitung

Die Wissensvermittlung im Rahmen des HNO-Blockpraktikums erfolgte durch ein vollständig digitales Curriculum. Praktische Fertigkeiten wurden mittels der Peyton-Lehrmethode vermittelt. Hierzu zählten die HNO-ärztliche Spiegeluntersuchung, Koniotomie und Kopf-Hals-Sonographie.

Das HNO-OSCE-Konzept wurde der Hygienekommission vorgelegt. Es erfolgte eine Begehung der Räumlichkeiten mit einem Hygienebeauftragten. Dieser genehmigte das HNO-Konzept, insbesondere aufgrund der Organisation einer stringenten zeitlichen Taktung und der kompletten Ausnutzung des HNO-Hörsaalgebäudes.

### Ablauf der Prüfung

**Figure 1:**
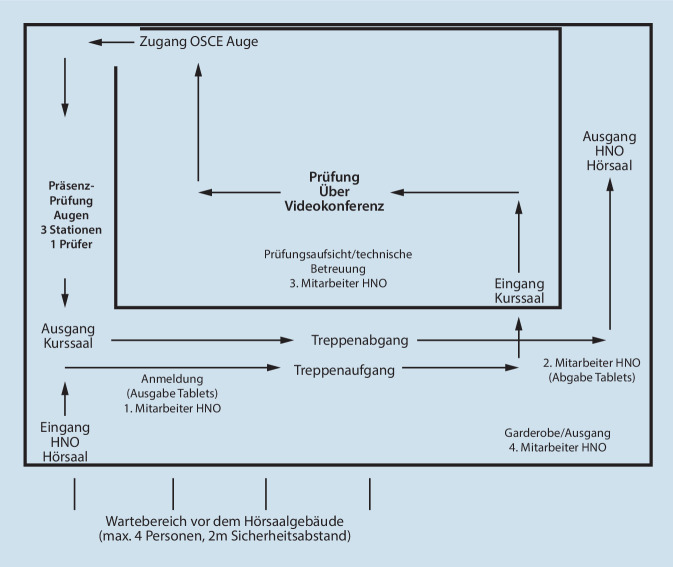

Insgesamt absolvierten 170 Studierende (*n* = 170) am Ende des Sommersemesters 2020 über 5 Tage verteilt ihre HNO- und Augen-OSCE. Pro Tag wurden etwa 36 Studierende in 3 Gruppen mit jeweils 12 Teilnehmern geprüft (Abb. 1).

**Figure 2:**
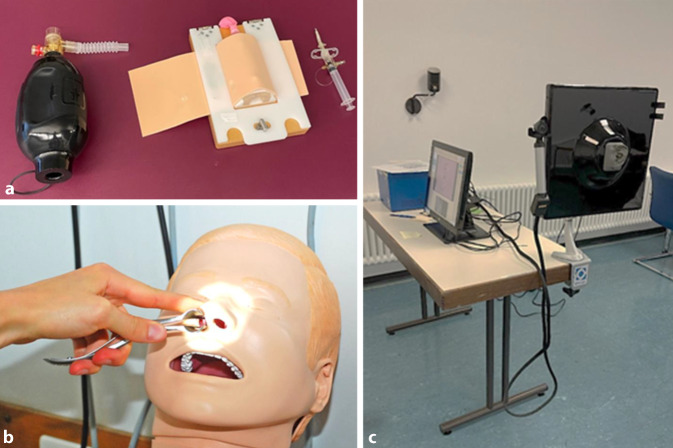

Die Prüfung fand in den Kurssälen des Hörsaalgebäudes der HNO- und Augenklinik statt, welche sich räumlich nebeneinander befinden. Alle Studierenden, Prüfer und studentischen Hilfskräfte waren zum Tragen von Mund-Nasen-Masken verpflichtet. Vor dem Hörsaalgebäude wurde ein Wartebereich für 4 Studierende eingerichtet mit einem Abstand von jeweils 2 m. An der Anmeldung, welche durch eine studentische Hilfskraft (Peer-Student) besetzt wurde, erfolgte die Ausgabe von Tablets. So konnten die Daten der Studierenden erfasst werden und den Prüflingen die Wartezeit mit Basisinformationen zur bevorstehenden OSCE bzw. zu den Rahmenbedingungen verkürzt werden. Mit Einhaltung des Sicherheitsabstands und entsprechender Wartezeit, auch auf dem Treppenaufgang, konnten die Tablets anschließend an einen Peer-Studierenden retourniert werden, bevor der Einlass durch einen weiteren Peer-Studierenden erfolgte. Dieser wies die Prüflinge ein (OSCE-Stationen an Puppen und Modellen) und war für die anschließende fachgerechte Desinfektion verantwortlich sowie für eine konstante Belüftung des Prüfungsareals. Diese Position wurde aus Sicherheitsgründen alle 45 min gewechselt. Nach Einweisung erfolgte an 3 OSCE-Stationen (Abb. 2a–c) die Prüfung über jeweils 3 min per Videokonferenz (Zoom). Die Prüfer waren ärztliche Mitarbeitende der HNO-Klinik und befanden sich in einem anderen Gebäude, konnten aber die 3 Stationen über die Webcam komplett einsehen. Im Anschluss an jede Station des Parcours erfolgte ein kurzes und prägnantes Feedback an die Studierenden über 1 min anhand von festgelegten Parametern (Kommunikation, Untersuchungstechnik/-ablauf, Faktenwissen).

Nach Abschluss der HNO-Prüfung erfolgte die Augen-OSCE als Präsenzprüfung (Prüfung an Puppen und Modellen, Anwesenheit eines Prüfenden) mit ebenfalls 3 Stationen.

### Bewertung der OSCE

Nach dem Abschluss beider OSCE erhielten die Studierenden einen Fragebogen zur Bewertung der beiden OSCE-Formate.

Zur Bewertung der OSCE wurden den Studierenden folgende Fragen und Antwortmöglichkeiten vorgegeben:

1. Welche OSCE-Variante fanden Sie besser?
  *Antwortmöglichkeiten:* Präsenz-OSCE (Auge), virtuelle OSCE (HNO), beide gleich.
2. Der Ablauf der kombinierten HNO-Auge-OSCE war …
  *Antwortmöglichkeiten:* sehr gut, gut, mittelmäßig, schlecht, sehr schlecht.
3. Das Prüfungsklima war insgesamt …
  *Antwortmöglichkeiten:* sehr angenehm, angenehm, mittelmäßig, unangenehm, sehr unangenehm.
4. Die Prüfung verschiedener OSCE-Stationen gemeinsam fand ich ….
  *Antwortmöglichkeiten: *sehr gut, gut, mittelmäßig, schlecht, sehr schlecht.
5. Das Feedback zur OSCE fand ich hilfreicher in der …
  *Antwortmöglichkeiten:* virtuellen OSCE (HNO), Präsenz-OSCE (Auge).
6. Die zur Verfügung gestellten Materialien waren bei der OSCE-Vorbereitung relevant.
  *Antwortmöglichkeiten:* trifft voll zu, trifft zu, unentschieden, trifft nicht zu, trifft gar nicht zu.
7. Ich bekam ausreichend Information zur OSCE-Vorbereitung.
  *Antwortmöglichkeiten:* trifft voll zu, trifft zu, unentschieden, trifft nicht zu, trifft gar nicht zu.
8. Nur HNO: Die Vermittlung mittels digitaler Peyton-Lehrmethode hat die Umsetzung ermöglicht.
  *Antwortmöglichkeiten:* trifft voll zu, trifft zu, unentschieden, trifft nicht zu, trifft gar nicht zu.

Zudem bestand die Möglichkeit, eine Bewertung in Form eines Freitexts zu hinterlassen.

### Ethische Gesichtspunkte

Die Studie wurde im Einklang mit nationalem Recht und der Deklaration von Helsinki in ihrer aktuellen Fassung von 2013 durchgeführt. Die Daten wurden anonym erhoben, ein Rückschluss auf die Teilnehmer war nicht möglich, weshalb eine Beratung durch die Ethikkommission der Albert-Ludwigs-Universität Freiburg nicht notwendig war.

## Ergebnisse

**Figure 3:**
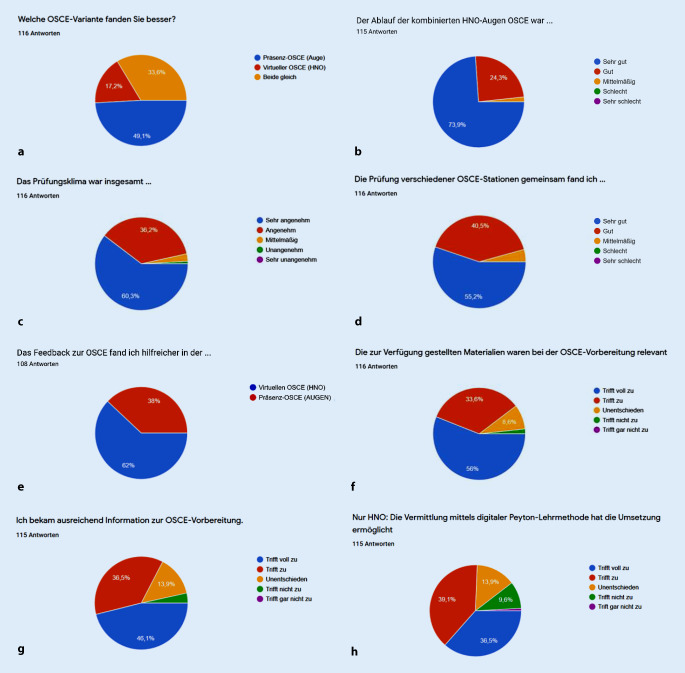

Zu jeder Evaluationsfrage erhielten die Autoren zwischen 108 und 116 Antworten zurück. Auf Frage 1 antworteten 49,1 % der Befragten, dass sie die Präsenz-OSCE (Auge) besser fanden, 33,6 % dass beide gleich waren, und 17,2 % fanden die virtuelle OSCE (HNO) besser (Abb. 3a). Den Ablauf der kombinierten HNO-Augen-OSCE (Frage 2) fanden 73,9 % der Befragten sehr gut, 24,3 % gut und 1,7 % mittelmäßig (Abb. 3b). Das Prüfungsklima (Frage 3) wurde mit 60,3 % als sehr angenehm, 36,2 % als angenehm, 2,6 % als mittelmäßig und mit 0,9 % als unangenehm angegeben (Abb. 3c). Die Prüfung verschiedener OSCE-Stationen gemeinsam (Frage 4) fanden 55,2 % sehr gut, 40,5 % gut und 4,3 % mittelmäßig (Abb. 3d). Das Feedback zur OSCE (Frage 5) fanden 62 % in der virtuellen OSCE (HNO) besser, 38 % in der Präsenz-OSCE (Auge; Abb. 3e). Die Frage, ob die zur Verfügung gestellten Materialien bei der OSCE-Vorbereitung relevant waren (Frage 6), befanden 56 % als voll zu treffend, 33,6 % als zutreffend, 8,6 % waren unentschieden, und 1,8 % empfanden es als nicht zutreffend (Abb. 3f). Als voll zutreffend, ausreichend Informationen zur OSCE-Vorbereitung erhalten zu haben (Frage 7), empfanden es 46,1 %, zutreffend fanden dies 36,5 %, dagegen waren 13,9 % unentschieden, und 3,5 % fanden dies nicht zutreffend (Abb. 3g). Die Frage, ob die Vermittlung praktischer Fertigkeiten mit der digitalen Peyton-Lehrmethode die Umsetzung der jeweiligen klinischen Tätigkeiten ermöglicht hat, beantworteten 39,6 % mit zutreffend, 36,5 % mit voll zutreffend, 13,9 % waren unentschieden, 9,6 % fanden es nicht zutreffend und 0,4 % gar nicht zutreffend (Abb. 3h).

Im Freitext des Feedbacks gab es v. a. die Kritik, dass das Audiosystem teilweise nicht zufriedenstellend funktionierte und dadurch die Prüfer in der HNO-OSCE mitunter nicht gut zu verstehen waren. Ein weiterer Kritikpunkt war, dass zwar die Prüfer nicht anwesend waren, jedoch studentische Hilfskräfte, was die Anzahl an Kontaktpersonen nicht vermindert hat. Bemängelt wurde auch, dass teilweise das praktische Üben in der Vorbereitung zu kurz kam. Es gab auch die Anmerkungen, dass alles super gewesen sei oder das Beste unter den Umständen erreicht wurde.

## Diskussion

Im Gegensatz zur Neuimplementierung eines Curriculums ist die Implementierung einer Prüfungssituation insgesamt als noch komplexer anzusehen, da sich dies im Extremfall zu einer justiziablen Angelegenheit ausweiten kann. Insofern müssen hier entsprechende Überlegungen sowohl in Richtung der Hygieneanforderungen als auch der inhaltlichen Leistungsüberprüfung und der rechtlichen Erfordernisse gehen. Insgesamt zeigten sich die Studierenden mit der Kombination der OSCE aus den Fächern HNO- und Augenheilkunde zufrieden. Hierbei wurde zwar die Präsenz-OSCE (Augenheilkunde) der virtuellen OSCE (HNO-Heilkunde) vorgezogen, wobei allerdings das Feedback an den OSCE-Stationen in der virtuellen HNO-OSCE als besser gewertet wurde. Das Prüfungsklima wurde insgesamt als angenehm empfunden. Im Gegensatz zu einer reinen Online-OSCE, wie in mehreren Studien untersucht [9, 14, 26, 28], erfolgte diese virtuelle Prüfung als Präsenzveranstaltung mit hohem logistischem und personellem Aufwand. Da virtuelle Prüfungssituationen bis dato in der HNO-Heilkunde gänzlich unbekannt oder zumindest nicht eindeutig definiert waren, erschien eine hybride Konstellation vorteilhaft. Zum einen ist eine Überprüfung der Einzelleistung gewährleistet, zum anderen kann hier Handlungswissen und nicht bloßes Faktenwissen, wie von Miller empfohlen, geprüft werden [18]. Somit kann mittels OSCE auch ein höheres Kompetenzlevel (Lernpyramide nach Miller) geprüft werden. Telemedizinische OSCE wurden auch schon vor COVID-19 beschrieben [2, 24], allerdings lag hier der Schwerpunkt auf einer telemedizinischen Patientenversorgung. In Singapur wurde bereits im März 2020 eine Präsenz-OSCE unter COVID-19 abgehalten, die Autoren kamen zu der Schlussfolgerung, dass es unter Pandemiebedingen möglich ist, ausreichend Maßnahmen zu ergreifen, um klinische Untersuchungen durchzuführen [1]. Diese Daten sind jedoch recht vage, nicht der HNO-Disziplin zugeordnet und zudem nicht anhand einer repräsentativen Zielgruppe evaluiert, wohingegen die vorliegenden Ergebnisse die genannten Kriterien erfüllen.

An der Universität Tübingen wurde im Oktober 2020 eine Präsenz-OSCE unter Pandemiebedingungen durchgeführt [16]. Auch hier zeigte sich, dass die Durchführung einer OSCE in Präsenz unter Pandemiebedingungen prinzipiell möglich ist. Die Studierenden fühlten sich allerdings durch das Tragen von medizinischen Masken in der Interaktion und nonverbalen Kommunikation mit den standardisierten Patienten eingeschränkt und hatten den Eindruck, dass es eher zu Missverständnissen kommen könne. Die Studierenden in der vorliegenden Erhebung machten demgegenüber hier viel präzisere Angaben und wiesen deutlich ihre Präferenzen auf.

Ob eine Präsenz-OSCE möglich ist, hängt im Wesentlichen von der Gesetzgebung ab, insbesondere davon, wie streng die Kontaktbeschränkungen ausgelegt werden. Allerdings ist es nicht ausreichend, nur die OSCE an die Pandemie anzupassen. Es muss das gesamte Curriculum angepasst werden, um den Studierenden eine digitale Lernumgebung und ein „constructive alignment“ zu schaffen, damit sich diese optimal auf die Prüfung vorbereiten können [16]. Insbesondere die Vermittlung klinisch-praktischer Fertigkeiten stellt eine Herausforderung dar. Hier wurde bereits in anderen Studien die erfolgreiche Anpassung verschiedener Methoden wie Peytons 4‑Schritt-Ansatz und Halsteds „See one, do one, teach one“ an eine partiell oder vollständig digitale Lehre beschrieben [12, 15, 25, 29]. In der vorliegenden Studie gaben 76,1 % der Studierenden an, dass es zutreffend oder voll zutreffend ist, dass die Vermittlung praktischer Fertigkeiten mit der digitalen Peyton-Lehrmethoden die Umsetzung der jeweiligen klinischen Tätigkeiten ermöglicht hat.

Auch wenn die digitale Lehre schon länger in unterschiedlichem Ausmaß existiert und auch bereits früher Aufforderungen zum Ausbau dieser bestanden, hat sie durch COVID-19 eine beschleunigte Entwicklung genommen. In Edinburgh wurde bereits 2003 eine erfolgreiche virtuelle Lernumgebung geschaffen, welche die Anwesenheitsveranstaltungen ergänzte [5]. Im Jahr 2011 beschrieb dieselbe Autorin, dass nach einem Jahrzehnt der Veränderung im Bereich des E‑Learnings wohl möglicherweise eine Phase der Konsolidierung erreicht wurde [4], welche nun ausgelöst durch die Kontaktbeschränkungen unter der Pandemie wieder durchbrochen scheint. Die Kombination aus virtueller Lehre und Präsenzlehre kann auch außerhalb einer Pandemiesituation das Curriculum sinnvoll ergänzen. Während der Pandemie gewonnene Erkenntnisse in der Lehre und etablierte virtuelle Strukturen sollten auch in Zukunft weiter genutzt werden [11, 27]. Insbesondere in Remote-Learning-Situationen wird es auch weiterhin notwendig sein, virtuelle Prüfungen durchzuführen.

Ein Kritikpunkt dieser Studie ist sicherlich, dass auch bei der virtuellen OSCE Hilfskräfte vor Ort sein mussten, um den Ablauf zu koordinieren, und es hier zu Kontakten kam. Zum Zeitpunkt der OSCE-Planung war die Situation der Pandemie aber noch relativ unübersichtlich. Es gab keine Modellprojekte, an denen man sich orientieren konnte, und keine oder zumindest wenig spezifische Vorgaben oder Hilfestellungen der medizinischen Fakultät. Mithilfe der eingesetzten Peer-Studenten war es möglich, die geltenden Hygienevorschriften bezüglich der Desinfektion aufrechtzuerhalten. Sie stellten zudem den zügigen Fluss der Prüfung sicher, um unnötige Kontakte durch Verzögerungen im Ablauf zu verhindern. Die von Seite der Autoren etablierten Wechselzeiten und die kontinuierliche Belüftung der Räumlichkeiten sowie die streng regulierte Organisation von Einzelpersonen zur Prüfung ermöglichte ein hohes und adäquates Maß an Sicherheit für die klinikeigenen Mitarbeiter und Studierenden.

Die Autoren führten eine rein subjektive Bewertung der OSCE durch die Studierenden durch. Eine objektive Prüfung des Erfolgs der Vermittlung der praktischen Fertigkeiten mit einem direkten Vergleich mit einer Kontrollgruppe von Studierenden, die eine traditionelle Präsenzvermittlung der praktischen Fertigkeiten erhielt, ist in dieser Studie nicht enthalten. Dies war ebenso wenig wie eine Randomisierung unter den gegebenen Hygieneverordnungen möglich. In früheren Studien wurden jedoch beide Vorgehensweisen (Peyton-Methode vs. traditionelle Lehrmethoden und virtuell vs. Präsenz) bereits als mindestens gleichwertig beschrieben [6, 13, 23].

Die Kombination aus HNO- und Augen-OSCE wurde aufgrund der Tatsache gewählt, dass die Augen-OSCE in Präsenz geplant wurde und somit der direkte Vergleich möglich wurde. Zudem teilen sich die HNO- und Augenklinik Räumlichkeiten für die Lehre. Ein Abgleich der Inhalte, insbesondere der geforderten Fertigkeiten, erfolgte nicht. Es ist denkbar, dass Unterschiede im Prüfungsniveau die Beurteilung des Prüfungsformats durch die Studierenden beeinflusst haben könnte.

Die Bewertung der Prüfungsleistung der Studierenden könnte die subjektive Beurteilung des Prüfungsformats ebenfalls beeinflussen. Dies kann durch das gewählte Studiendesign nicht ausgeschlossen werden. Die grundsätzlich positive Bewertung durch die Studierenden schließt dies jedoch weitestgehend aus.

Zusammenfassend zeigte sich eine gute Durchführbarkeit der Kombination aus HNO- und augenheilkundlicher OSCE, wobei die Studierenden die Präsenz-OSCE der virtuellen OSCE vorgezogen. Bei insgesamt hoher Zufriedenheit auf studentischer Seite zeigt sich die Machbarkeit einer virtuellen Prüfung, welche einer detaillierten und gut geplanten Vorbereitung bedarf. Hierfür müssen nicht nur die Prüfer gründlich eingewiesen werden, auch die Prüflinge müssen speziell hierauf vorbereitet werden. Um dies zu erreichen, war die Umstellung auf ein digitales Curriculum Voraussetzung.

## Fazit für die Praxis

- Unter Pandemiebedingungen ist eine virtuelle („objective structured clinical examination“) OSCE möglich.
- Prüfer und Prüflinge müssen speziell darauf vorbereitet sein, u. a. ist hierfür ein digitales Curriculum Voraussetzung.
- Studierende bevorzugen eine Präsenz-OSCE gegenüber einer virtuellen OSCE.
